# Improved Mechanical and Thermal Properties of Date Palm Microfiber-Reinforced PCL Biocomposites for Rigid Packaging

**DOI:** 10.3390/molecules30040857

**Published:** 2025-02-13

**Authors:** Sakib Hossain Khan, Hom N. Dhakal, Abu Saifullah, Zhongyi Zhang

**Affiliations:** Portsmouth Centre for Advanced Materials and Manufacturing (PCAMM), School of Electrical and Mechanical Engineering, University of Portsmouth, Portsmouth PO1 3DJ, UK; sakib.khan@port.ac.uk (S.H.K.); abu.saifullah@port.ac.uk (A.S.); zhongyi.zhang@port.ac.uk (Z.Z.)

**Keywords:** date palm fibers, mechanical properties, biocomposites, surface treatment, packaging

## Abstract

To increase the use of agricultural residues, such as date palm fibers, for the sustainable reinforcement of biocomposites, this study investigated the incorporation of varying weight percentages of date palm microfibers (DPMF) ranging from 0 wt.% to 10 wt.% into polycaprolactone (PCL) matrix. Biocomposites were fabricated using a combination of compression molding and dry blending techniques with and without sodium hydroxide (NaOH) alkali treatment. The surface modification was found to increase the surface roughness of the fibers, removing impurities such as lignin, hemicellulose, and wax, while improving crystallinity, as evidenced by FTIR, XRD, TGA, and particle size analyses. Among the different biocomposites investigated, the results for 5 wt.% DPMF content biocomposites exhibited the highest tensile properties: approximately 20% increase in tensile strength and 164% increase in Young’s Modulus in comparison to neat PCL. The crystallinity of the matrix exhibited an increasing trend from approximately 39% for neat PCL to 43% for the 5 wt.% DPMF biocomposites. Furthermore, treated biocomposites demonstrated higher water-repellency behavior and improved thermal properties. Dynamic mechanical analysis (DMA) results indicated enhanced storage moduli for alkali-treated composites; at 35 °C, the storage modulus showed approximately 22% increase compared to the untreated DPMF biocomposites, reflecting improved stiffness and thermomechanical performances. This study highlights the potential of DPMF as an efficient, eco-friendly alternative to fossil-based conventional reinforcement for biocomposite materials’ potential for sustainable rigid packaging applications.

## 1. Introduction

The date palm (*Phoenix dactylifera* L.), also known as the “tree of life”, is widely available, especially in North Africa and the Middle East, where it is extensively cultivated for its fruit and holds significant medicinal, nutritional, and economic values in these regions. Each date palm tree produces a substantial amount of agricultural residues annually, with a typical tree generating around 40 kg of waste [1]. These residues come from various parts of the tree, including dried leaves, sheaths, and petioles. Date pits, which account for nearly 10% of the fruit, also contribute to this biomass. In total, some countries like Saudi Arabia generate more than 200,000 tons of date palm biomass each year. Currently, most of these residues are either burned on farms or disposed of in landfills, leading to significant environmental pollution [2].

Major agricultural residues are the by-products left after the harvesting and processing of crops. These residues can be classified into two categories: field residues and processing residues [3]. The field residues include straw, which remains after harvesting grains like wheat, rice, barley, and oats, and the processing residues, which include fruit and vegetable peels and sugarcane bagasse [4,5]. Date palm residues fall into both field residues and processing residues categories, such as palm fronds, which are the leaves of the date palm tree that are regularly pruned during cultivation, and date seeds (pits), which are the seeds left after processing the dates for food [6].

**Figure 1:**
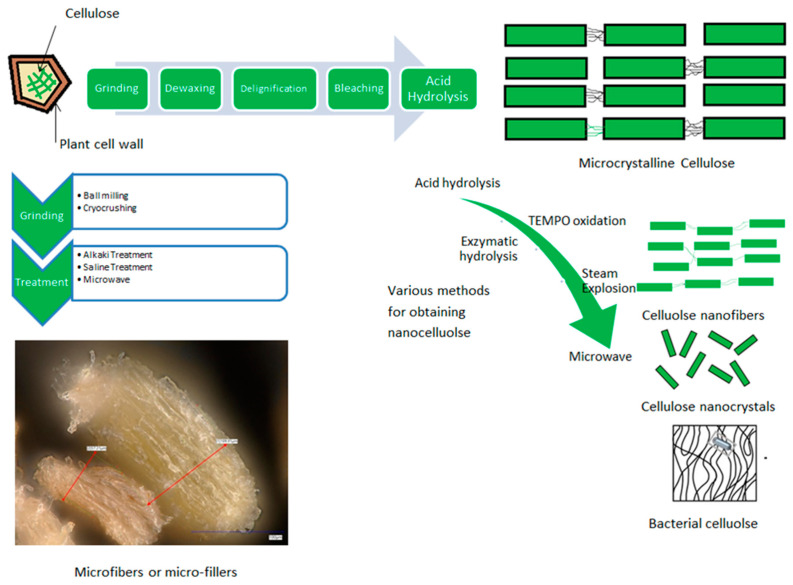
Steps involved in extraction of cellulosic materials from agricultural residues.

Date palm agricultural residues are a rich source of cellulose, making them valuable for sustainable product development [7]. Various parts of the date palm tree, including fronds, rachis, and bunch stalks, contain significant amounts of cellulose, ranging from 21% to 44%. These residues, which are typically left behind after harvest, have considerable potential for producing bio-products which are increasingly used in environmentally friendly packaging materials. Recent research highlighted the cellulose content of date palm waste residues, revealing that they consist of 34% cellulose, along with hemicellulose and lignin, further confirming the potential of these residues for various structural and semi-applications [8]. Environmental concerns associated with improper disposal of date palm biomass, particularly bunch stalks and leaflets, can be mitigated by converting them into high-value products like micro- or nano-cellulose [9], as illustrated in Figure 1. The cellulose content of these residues can contribute to the development of sustainable packaging both as micro- or nano-filler and as a film-forming agent, offering benefits such as biodegradability and lower environmental impact compared to traditional plastic-based packaging [10].

Agricultural residues offer valuable resources for the production of biopolymers and biocomposites, functioning as sources of monomers, additives, and fillers, as illustrated in Figure 1. Their integration into material development provides eco-friendly alternatives to traditional plastics while facilitating waste recycling and advancing circular economy initiatives. This strategy mitigates environmental pollution, reduces waste disposal costs, and decreases reliance on synthetic polymers, enabling the cost-effective production of biodegradable materials. The feasibility of utilizing agricultural residues in biopolymer and biocomposite fabrication has been extensively demonstrated. For example, rigid packaging biocomposites have been successfully developed using byproducts such as mango seeds blended into PLA matrices through extrusion and injection molding, with waste content reaching up to 20% [11]. Composite films incorporating rice straw and nanofibers as reinforcements in chitosan-based polymer systems have also shown promise for sustainable packaging applications [12]. Additionally, bio-packaging films derived from shallot stalks and tamarind seeds present viable alternatives to conventional plastic films [13]. These examples highlight the potential of agricultural residues, including mango seeds, rice straw, and onion waste, as economical fillers that improve the sustainability and functional properties of packaging materials [14].

Agricultural residues like date palm wastes can be optimized using various extraction methods, as illustrated in Figure 1, to obtain valuable components such as cellulose [15], micro-fibrillated cellulose [16], microcrystalline cellulose [17], nanofibers [9], crystalline nanocellulose [18], all of which have a particularly promising prospect in packaging application. The selection of the optimal extraction method depends on the specific physical and chemical characteristics of residues. The widespread use of conventional extraction techniques is largely due to their ease and efficiency. Conventional chemical extraction methods include acid or alkaline reagents or solvents, but these methods generally provide low yield, increase the handling process for removing the residuals, and raise considerable concern regarding the disposal of the solvents and reagents after the completion of the process [5,19]. These methods can be combined with other novel methods to maximize the yield and reduce chemical waste. The novel methods to extract cellulose and cellulose derivatives include microwave, ultrasonication, steam explosion, and ball milling, but these are associated with high energy consumption [5]. Thus, a combination and optimization of these novel methods and chemical extraction processes aligns with the principles of “green” extraction techniques [19]. The microwave-assisted extraction of MCC from walnut shell, corncob, and sugarcane bagasse is reported to produce high yield and purity and requires fewer chemicals compared to other processes [20]. Traditionally, cellulose is obtained from lignocellulosic agricultural residues through acid–alkali treatments that remove non-cellulosic fractions like hemicellulose and lignin. This involves the use of chemicals like sodium hydroxide and bleaching agents to isolate cellulose, a process that has been successfully applied to date palm waste, resulting in high-quality cellulose [8]. In recent years, alternative methods like low-alkali treatments and the use of rejected brine from desalination plants have been explored to reduce the environmental impact of cellulose extraction [15]. Brine, with its high chloride ion content, helps break down lignin and enrich cellulose while reusing waste materials in a sustainable manner [21]. Once extracted, cellulose can be further processed into microcellulose or nanocellulose, each with unique properties and applications [22].

**Figure 2:**
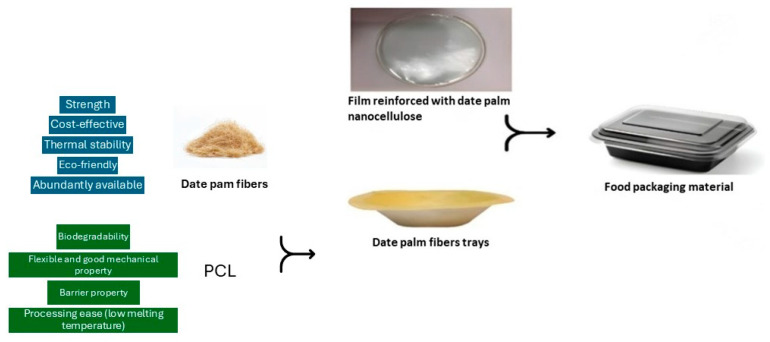
Suitability of DPMFs and PCL in packaging application.

Date palm micro-fibers have also demonstrated considerable potential as reinforcement for polymeric materials for packaging applications, as displayed in Figure 2, particularly following chemical treatment processes. Nassar et al. employed cryogenic ball milling for the preparation of date palm micro-fibers, followed by a systematic chemical treatment comprising dewaxing, acetylation, and mercerization, all conducted under controlled microwave heating conditions [23]. When these treated fibers are used as reinforcement in PP, they significantly improve the composite’s mechanical properties. The treatment boosts the interfacial adhesion between the fibers and the polymer through chemical crosslinking, resulting in increased tensile strength, flexural strength, and stiffness. In the work conducted by Mousa and colleagues, date palm rachis (DPR) microfibers were employed as natural fillers within a polylactic acid (PLA) matrix to develop biodegradable composites for packaging applications, particularly for single-use plastics such as cutlery [24].

**Figure 3:**
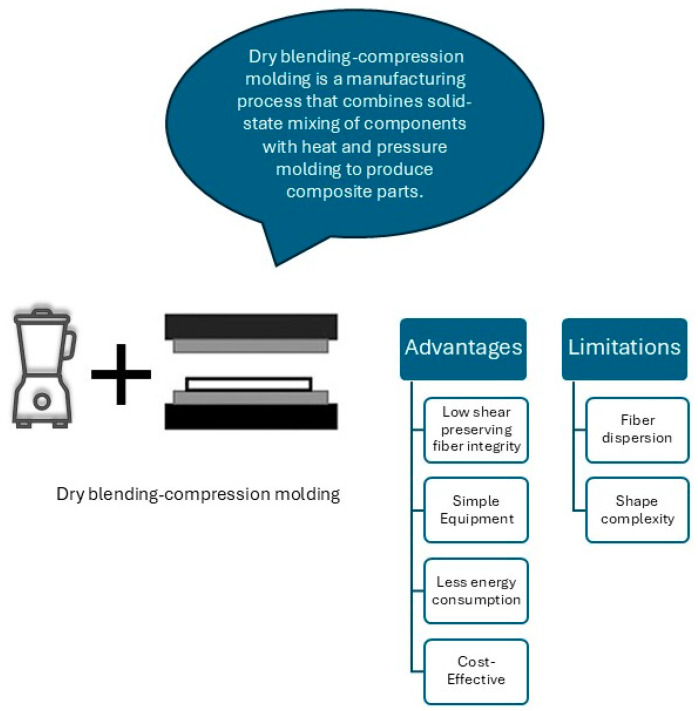
Dry blending–compression molding benefits.

Dry blending is an effective technique for producing lab-scale samples of biocomposites due to its simplicity and ease of operation, as illustrated in Figure 3 [25]. Using the microfibers obtained from date palm leaf, Saifullah et al. [26] used the dry blending method, which requires lower energy compared to the melt mixing and injection molding process employed by Mousa for DPR–PLA composites, primarily due to distinct variations in processing techniques and operating temperatures. While the melt mixing of DPR–PLA composites occurs at 180 °C, Saifullah et al. utilized a compression molding process to fabricate composites using polycaprolactone (PCL) reinforced with date palm microfibers at a relatively low processing temperature of 120 °C [26]. PCL, a biocompatible biopolymer, is widely used in biomedical and packaging applications due to its low melting temperature and ease of processing [27], making it particularly suitable for compression molding techniques. The melt mixing process for PLA composites typically involves continuous shear and high-pressure conditions. In this work, the fibers and polymers are grounded into micro-particles to achieve a uniform blend, with air pockets effectively eliminated. Dry blending was chosen for DPMF-PCL biocomposites due to its simplicity, cost-effectiveness, and suitability for energy-efficient lab-scale production and rapid prototyping.

There is considerable potential for utilizing date palm residues in environmentally friendly applications, including the development of packaging materials. Date palm fibers have been studied for various uses, such as packaging, construction, and automotive [28,29]. The key objective of this work is to develop fully biodegradable biocomposites with the use of bio-fiber, such as date palm and PCL, as an environmentally friendly material and investigate their key properties for rigid packaging applications. For this, a dry-blending process was used where alkali-treated and untreated date palm microfibers were used and compared as reinforcements with an aim to develop fully biodegradable light-weight biocomposites to replace fossil-based polymer and composites largely being used in various sectors, including in packaging applications.

## 2. Materials and Methods

### 2.1. Materials

Date palm leaf fibers were sourced from Saudi Arabia. To eliminate impurities, the fibers were thoroughly washed with tap water and then dried in an oven at 85 °C for 24 h. PCL used in this project was supplied by Easy Composites, Stoke on Trent, U.K. (Approximate Molecular Weight (Mw): 55,000 (determined by GPC), Specific Gravity: 1.145 (at 23 °C), Melting Point: 60 °C, Melt Flow Index (MFI): 9 g/10 min (at 80 °C, 2.16 kg load), Glass Transition Temperature (Tg): −60 °C, Decomposition Temperature: 200 °C, CAS Number: 24980-41-4, Purity: >99%)

### 2.2. Methods

#### 2.2.1. Microfiber Extraction and Fiber Treatment

**Figure 4:**
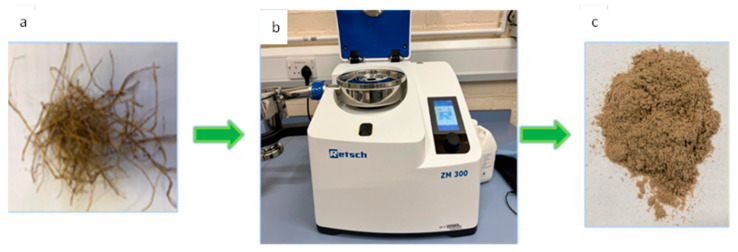
Fiber-grinding process: (**a**) date palm leaf fibers; (**b**) RETSCH Ultra Centrifugal Mill ZM 300; (**c**) date palm micro-fibers.

The washed and oven-dried fibers are cut into 10 mm segments and then processed using an Ultra Centrifugal Mill ZM 300 (Retsch, Germany) in a cryogenic environment to achieve a microfiber scale, as shown in Figure 4. The milling process is carried out at a rotational speed of 14,000 rpm, utilizing a sieve with a 0.25 mm mesh size.

The ground fibers were subjected to alkalization by immersing them in a 5 wt.% NaOH solution for 2 h at room temperature. A 5% concentration was selected based on literature findings [30,31,32,33], which indicate that this concentration yields the maximum cellulose content after treatment. After the treatment using 5 wt.% NaOH, the resulting fibers are washed with distilled water 5 times to remove the residuals from the surface of the fibers. The treated fibers are placed in an oven for 24 h at 95 °C to remove the storage moisture.

#### 2.2.2. Biocomposites Fabrication

Poly(ε-caprolactone) (PCL) pellets and date palm microfibers were used in this study. To achieve similar particle sizes and improve blend homogeneity, PCL pellets were cryogenically ground using liquid nitrogen to prevent clogging of the grinder blades and minimize thermal degradation. This process yielded fine PCL powders suitable for the dry-blending process. The PCL polymer powder was blended with date palm microfibers in various weight ratios using a magnetic shear mixer. This dry blending technique was employed to achieve a uniform dispersion of the microfibers within the polymer matrix and to minimize agglomeration and void formation, which were observed in previous work when pellets were used. The dry-blended PCL/date palm microfiber mixtures were then subjected to compression molding.

**Figure 5:**
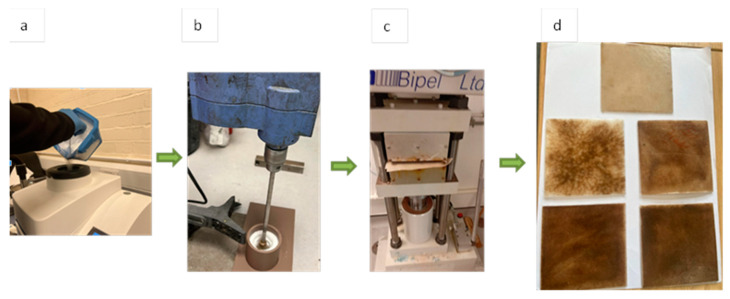
Description of dry-blending process used for biocomposites fabrication: (**a**) polymer grinding; (**b**) shear mixing of fibers and polymer; (**c**) compression molding; (**d**) final biocomposites plates.

The molding process was conducted using an electrically heated hydraulic press, as shown in Figure 5, following these steps:

The empty mold, including both the upper and lower plates, was preheated to 140 °C for 40 min to ensure uniform temperature distribution. Then, the polymer/fiber mixture was quickly and evenly spread onto the preheated lower mold plate. After that, the mold was closed and subjected to an additional heating stage at 140 °C for 60 min under a pressure of 10 MPa to promote adequate fusion and consolidation of the mixture. Finally, the pressure was released, and the mold was allowed to cool for 2 h at room temperature to ensure complete solidification of the molded plaques. After cooling, the plaques were carefully demolded to obtain 90 mm × 90 mm × 2 mm samples.

## 3. Characterization Techniques

### 3.1. Micro-Fiber Characterization

Untreated and treated surfaces of date palm micro-fibers were characterized using FTIR, XRD, optical microscope, TGA, and particle size analyzer.

#### 3.1.1. Fourier Transform Infrared Spectroscopy (FTIR)

Fourier Transform Infrared Spectroscopy (FTIR) analysis was performed at the University of Portsmouth. Both NaOH-treated and untreated fibers were ground into a fine powder and analyzed using a Thermo Nicolet Nexus 870 ESP FT-IR spectrometer (Spectralab Scientific Inc., Markham, ON, Canada) equipped with an Attenuated Total Reflectance (ATR) accessory (Bruker, Billerica, MA, USA). The spectra were recorded over a range of 500 to 4000 cm^−1^ with a resolution of 2 cm^−1^, and each sample underwent 64 scans to ensure accurate spectral data acquisition.

#### 3.1.2. X-Ray Diffraction (XRD) Analysis

The X-ray diffraction (XRD) analysis of the finely chopped fiber and composite samples was performed using a compact Aeris X-ray diffractometer manufactured by Malvern Panalytical, operating at 40 kV and 15 mA. The crystallinity index was calculated by the formula after performing the baseline correction using the Equation (1):

(1) $$Crystallinity\;index=\frac{I_{total}-I_{amorphous}}{I_{total}}$$

In Equation (1), *I_total_* = Total area, and *I_amorphous_* = Area under the amorphous region.

The Scherrer Equation (2) was used to calculate crystallite size (*CS*) [34]:

(2) $$CS=\frac{k\lambda }{\beta \cos\theta }$$

In Equation (2), *k* is the Scherrer constant (0.9), *λ* is the X-ray wavelength (0.154 nm), *θ* is the Bragg angle, and *β* is the peak full width at half maximum (FWHM).

#### 3.1.3. Optical Microscopy Examination

The physical characterization of the untreated and the treated was made through the use of a VHX digital optical microscope (Digital Microscope VHX-7000 Series|KEYENCE International Belgium, Mechelen, Belgium). From the optical microscope analysis of the date palm untreated and treated microfibers, we can obtain detailed images that allow us to examine the surface and measure the diameters and lengths of the individual fibers.

#### 3.1.4. Thermogravimetric Analysis (TGA)

TA Q 50 TGA (TA Instruments, New Castle, DE, USA) equipment was used to investigate the degradation behavior of alkali-treated and untreated date palm microfibers in an air atmosphere in the temperature range from 30 °C to 600 °C at a heating rate of 20 °C/min.

#### 3.1.5. Particle Size Analysis

A particle size analysis of untreated and treated date palm microfibers was conducted using a Sympatec HELOS/BF laser diffractometer (Clausthal-Zellerfeld, Germany) coupled with a RODOS dry dispersion system. For each measurement, approximately 5 mg of the sample was dispersed through a laser beam (wavelength: 875 nm) at an air pressure of 1 bar. The analysis employed a 100 mm lens, with calculations based on the Fraunhofer diffraction theory.

The particle size distribution was characterized by the volume-based percentiles d10, d50, and d90, representing particle diameters at 10%, 50%, and 90% cumulative volume, respectively. The volume median diameter (VMD), corresponding to the d50 value, was also recorded, indicating the median particle size by volume. This methodology ensures reliable and accurate characterization of fiber size and distribution, enabling a comparative assessment of untreated and treated microfibers.

### 3.2. Biocomposite Characterization

#### 3.2.1. Tensile Testing

**Figure 6:**
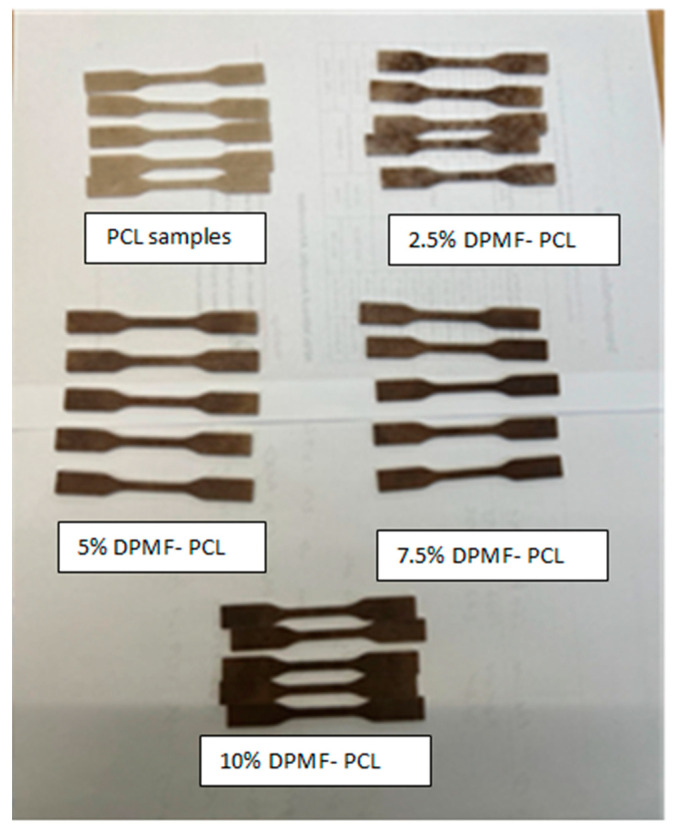
The neat PCL and different concentrations of DPMF composites where the samples became darker with the increase in micro-fiber content compared to the neat PCL.

A tensile test was performed on biocomposite samples made of polycaprolactone (PCL) and date palm microfibers, as shown in Figure 6, in accordance with the ASTM D638-14 standard [35]. The tests were carried out using a Zwick/Roell Z010 (Ulm, Germany) universal testing machine equipped with a 10 kN load cell. Type V specimens, prepared using a laser cutter, were tested at a constant displacement rate of 50 mm/min until failure. A total of five samples were tested for each biocomposite type.

#### 3.2.2. Flexural Testing

The flexural properties, including flexural strength and modulus, of the date palm fiber-reinforced polycaprolactone (PCL) composites were evaluated through standardized flexural testing. Test specimens were prepared in a rectangular geometry according to the ASTM D790 protocol [36] The flexural test was conducted using a Zwick/Roell Z010 (Ulm, Germany) universal testing machine, equipped with a 10 kN load cell and operating at a constant crosshead speed of 20 mm/min. The test span was kept at 50 mm.

#### 3.2.3. Study of Moisture Absorption Behavior

The date palm microfiber-reinforced PCL composite panels were subjected to accelerated aging under high humidity conditions within an environmental chamber. The chamber temperature was maintained at 30 °C, which is marginally above ambient temperature, as PCL’s low melting point precludes the use of higher temperatures. During the moisture aging process, the composite laminates were continuously monitored for weight variations to quantify the moisture uptake. The experimental procedure adhered to ASTM D5229 standards [37] for moisture absorption testing on both alkali-treated and untreated date palm microfiber composites. The test was concluded once the composite’s moisture content stabilized, indicating that the material had reached its saturation point. The moisture content was then calculated using the Equation (3):

(3) $$Mositure\;Absorption,\;M_{t}=\frac{W_{t}-W_{0}}{W_{0}}$$

In Equation (3), *W*_0_ = original mass of the composite specimen, and *W_t_* = mass taken at time t.

#### 3.2.4. Water Contact Angle Measurement

The water contact angle of biocomposite surfaces was measured using a KSV CAM-101 (KSV Instruments Ltd., Espoo, Finland) instrument paired with CAM 2008 software to assess the wettability of alkali-treated and untreated date palm microfiber-reinforced PCL biocomposites. Water droplets were deposited at various locations on each sample, five readings were recorded, and an average contact angle value was calculated based on five measurements per sample.

#### 3.2.5. Dynamic Mechanical Analysis (DMA)

The thermo-mechanical properties of biocomposites were evaluated using a Q800 dynamic mechanical analyzer (TA Instruments, New Castle, DE, USA) under double cantilever mode. The tests were conducted in air over a temperature range of 20 °C to 50 °C, with a heating rate of 2 °C/min. A 1 Hz oscillation frequency and a strain of 0.01% were applied during the measurements. Samples were prepared in a cuboid shape with dimensions of 80 mm × 10 mm × 2 mm. The experiments provided data on both the storage modulus (E′) and loss modulus (E″). 

#### 3.2.6. Differential Scanning Calorimeter (DSC) Analysis

Thermal characterization of the materials was conducted using a differential scanning calorimeter (DSC) (TA Q 100 DSC equipment, New Castle, DE, USA). Pre-weighed samples (~6 mg) were subjected to heating and cooling cycles ranging from −50 °C to 80 °C at a scanning rate of 10 °C/min under a nitrogen atmosphere. The degree of crystallinity of PCL (χ) was calculated using the following equation (Equation (4)):

(4) $$\chi \left(\%\right)=\frac{∆H_{m}}{∆H_{PCL}^{0}\times X_{PCL}}$$

In Equation (4), $∆H_{m}$ = melting enthalpy of the samples, $X_{PCL}$ = weight fraction of PCL in the composite, and $∆H_{PCL}^{0}$ = 100% crystalline PCL melting enthalpy (139.5 J/g) [38].

#### 3.2.7. Rheology Measurements

Rheological properties of the polymer melts were analyzed using a Discovery Hybrid 3 rotational rheometer (TA Instruments, New Castle, DE, USA) configured with a parallel-plate geometry of 25 mm diameter. Frequency sweep tests were conducted at a temperature of 140 °C over a range of frequencies from 0.01 to 100 Hz for neat PCL, 5 wt.% DP microfiber–PCL, and the 10 wt.% of DP microfiber–PCL. PCL-based specimens for rheological evaluation were prepared through compression molding, forming discs with a 25 mm diameter. The flow curve is also obtained for neat PCL, 5% DP microfiber–PCL, and the % of DP microfiber–PCL at 140 °C.

#### 3.2.8. Morphological Characterization of Fractured Region

The surface morphology of the tensile fractured region of the treated and untreated DP microfiber–PCL composites was characterized using a Zeiss Evo 10 scanning electron microscopy (SEM), Carl Zeiss Microscopy GmbH (Jena, Germany). All samples were coated with gold before SEM analysis.

## 4. Results and Discussion

The results and discussion section comprehensively analyze the surface properties, structural changes, thermal stability, surface morphology, and particle size of raw and alkali-treated date palm microfibers, providing valuable insights into their potential as a reinforcement in polymeric composites.

The results and discussion section delve into the mechanical, thermal, rheological, and morphological properties of biocomposites comprising date palm micro-fibers, both untreated and alkali-treated, incorporated into PCL polymers at varying percentages (0%, 2.5%, 5%, 7.5%, and 10%), to evaluate their suitability for rigid packaging applications.

### 4.1. FT-IR Analysis

**Figure 7:**
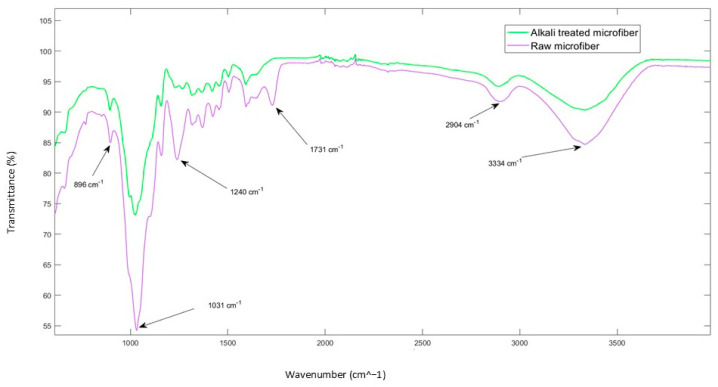
FTIR spectra for untreated and treated DP microfibers.

Alkaline treatment of lignocellulosic fibers removes non-cellulosic components, increasing fiber density and potentially enhancing mechanical properties [39]. Figure 7 illustrates the FTIR spectra for date palm microfibers treated with 5 wt.% NaOH for two hours. The prominent peak at 1031 cm^−1^, corresponding to cellulose, remains evident post-treatment, signifying the preservation of cellulose structure [40]. However, the disappearance of the peak at 1240 cm^−1^ suggests the complete removal of lignin [41]. Similarly, the absence of the 1731 cm^−1^ peak indicates that hemicellulose and pectin have been effectively removed following alkali treatment [42]. This confirms the efficacy of the alkali treatment in selectively degrading hemicelluloses, lignin, and pectin, leaving behind a cellulose-rich fiber.

Fourier-transform infrared spectroscopy (FTIR) provides a rapid and qualitative assessment of alterations in chemical structure. The FTIR spectra for both untreated and NaOH-treated date palm microfibers are shown in Figure 5. A broad absorption band near 3334 cm^−1^ corresponds to hydrogen-bonded O–H stretching, which appears in both spectra. Additionally, a peak around 2904 cm^−1^ is attributed to aliphatic C–H stretching vibrations. A notable feature in the spectra is the appearance of a strong peak at 1031 cm^−1^, which corresponds to the asymmetric stretching of C–O–C bonds in cellulose. The band at 1240 cm^−1^, associated with the C–O stretching, signifies the presence of lignin within the fiber. Moreover, the peak observed at 1731 cm^−1^ is attributed to C=O stretching, indicative of hemicelluloses and pectin content.

### 4.2. XRD Analysis

Alkali treatment has been shown to enhance the crystallinity index of date palm leaf microfibers. When treated with 5% sodium hydroxide (NaOH) for 2 h, the crystallinity and crystallinity index of the fibers increased. The crystallinity index, calculated using Equation (1), was found to be 71.7% after the treatment.

**Figure 8:**
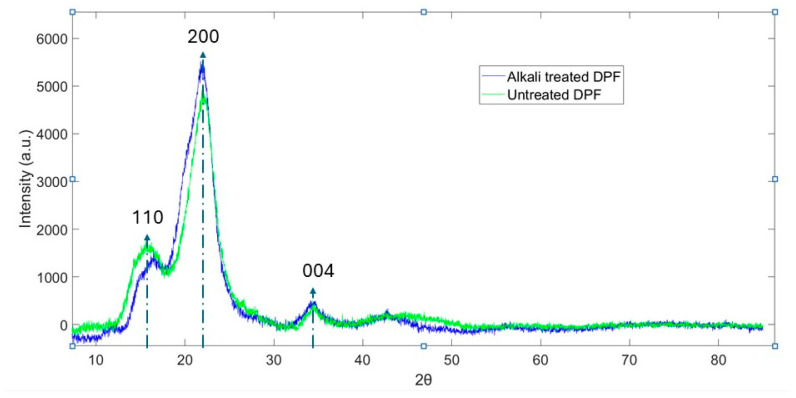
X-ray diffraction (XRD) spectra of untreated and treated DP microfibers.

In untreated fibers, as shown in Figure 8, the intensity of the crystalline peak (I_22_), which was initially 4867, rose to 5422 after alkali treatment after baseline correction, signifying an increase in the crystallinity index compared to raw fibers. A decrease in crystallite size was observed following the surface treatment of date palm microfibers. The average crystallite size (CS) for untreated fibers was calculated using the Scherrer equation, and the average value was 1.95 nm, while the treated fibers exhibited a reduced size of 1.88 nm. These findings are in close agreement with the results reported by Amior et al. [34]. The primary mechanism driving this improvement is the removal of non-cellulosic materials such as lignin, wax, hemicellulose, and amorphous cellulose during alkali treatment [39], and this is also evident in our FTIR results where peaks 1240 cm^−1^ and 1731 cm^−1^ are not present in the treated samples. These components contribute to the amorphous behavior of the untreated fibers, reducing their crystallinity [43]. As these non-crystalline materials are eliminated, the cellulose chains become more orderly and better aligned, resulting in an increase in the crystallinity index of the micro-fibers.

**Figure 9:**
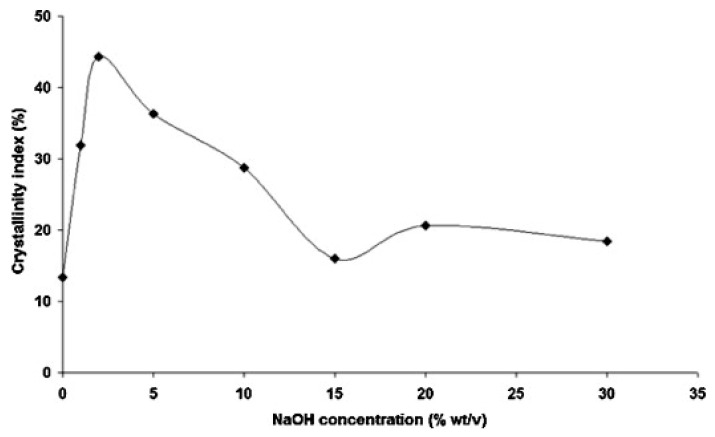
Crystallinity index versus NaOH concentration for agave fibers [44] (Copyright License number: 5956470259977).

The process involves NaOH penetration into the amorphous regions, causing swelling and transforming native cellulose I (parallel chain alignment) to cellulose II (anti-parallel alignment) through intermediate soda cellulose phases [44]. As a result, the crystallinity index (CI%) initially increases, peaking at around 5% NaOH concentration due to the effective removal of non-cellulosic materials and improved chain alignment [45]. However, at concentrations above 6%, the excess penetration of Na^+^ ions leads to over-swelling and disruption of the crystalline structure, as shown in Figure 9, resulting in polymorphic transformations, reduced crystallinity, and fiber degradation [46,47]. The optimal concentration was found to be 5%, where the balance between structural enhancement and mechanical integrity is maintained, as observed in fibers like banana, agave, and bamboo [44,48,49]. The increase and decrease in crystallite size were also governed by the same mechanism described for crystallinity index.

### 4.3. Thermogravimetric Analysis (TGA) Results

**Figure 10:**
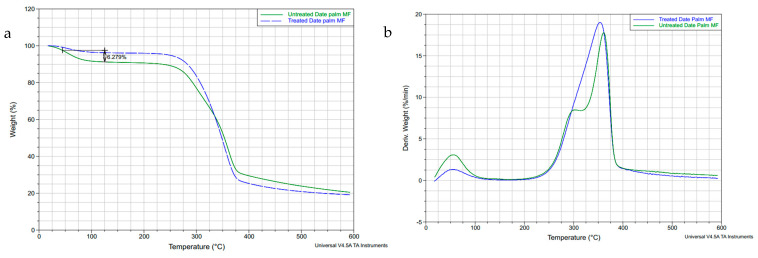
Thermogravimetric Analysis (TGA): (**a**) weight loss versus temperature traces; (**b**) derivative thermogravimetric (DTG) of untreated and alkali-treated date palm microfibers.

The TGA analysis results of the treated and untreated date palm micro-fibers are presented in Figure 7, with a more detailed depiction of the degradation process shown in the derivative thermogravimetric (DTG) graph in Figure 10b. The DTG graph illustrates the rate of weight loss (%/min) during the thermal degradation of date palm fibers. Untreated date palm microfibers began losing weight at a lower temperature, below 100 °C, likely due to their higher moisture content, which can be attributed to the presence of hemicellulose. Similar findings have shown that untreated fibers experience greater initial weight loss from moisture evaporation compared to NaOH-treated fibers [50].

The DTG curve in Figure 10b demonstrates different degradation patterns for treated and untreated fibers. Untreated fibers showed a two-step degradation process, with the first peak occurring around 290 °C, corresponding to the breakdown of hemicellulose and parts of lignin [51]. In contrast, treated fibers exhibited a single-step degradation, with no weight loss at 290 °C, which is explained by the removal of hemicellulose during the alkalization process. There was a slight difference in the residue left after heating to 500 °C, with alkaline-treated fibers leaving fewer residues than untreated ones. This suggests that the chemical treatment improves the thermal stability of the fibers by eliminating unstable constituents like hemicellulose and lignin.

### 4.4. Particle Size Analysis Results

**Figure 11:**
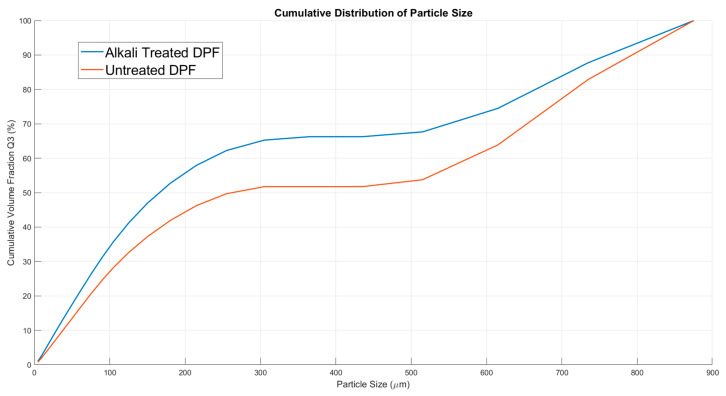
Cumulative distribution of the particle size of the untreated and treated date palm micro-fibers.

Alkali treatment significantly impacts the particle size distribution of date palm microfibers by reducing the particle sizes and making the distribution more uniform [51]. This is evident from the d10, d50, and d90 percentiles, which indicate the size below which 10%, 50%, and 90% of the particles lie, respectively. For alkali-treated fibers, d10, d50, and d90 values are 29.22 µm, 166.02 µm, and 760.62 µm, compared to 37.89 µm, 262.35 µm, and 793.13 µm for untreated fibers as illustrated in Figure 11. This reduction in particle size occurs because alkali treatment breaks down fiber bundles into smaller fibers and removes surface components such as lignin, hemicellulose, wax, and oils.

The treatment smoothens the fiber surface, indicating the residual matters were eliminated, as evident in our FTIR, XRD, and TGA analysis, which further contributes to a smaller and more uniform particle size [52]. This process exposes more surface area and creates rougher topography due to the removal of surface impurities. Such changes enhance the properties of the fibers, making them more suitable for applications like rigid packaging that require high aspect ratio particles and better interaction with matrices in composite materials.

### 4.5. Fiber Morphology Using Optical Microscope

Microscopic images of the plant fibers provide critical insights into the microstructural and morphological characteristics of the materials. They reveal surface textures, the presence of impurities, and structural changes induced by chemical treatments. For date palm fibers, microscopic images show a polygonal to round morphology with a distinctive central void [53], which influences their mechanical properties and interaction with polymer matrices in composite applications. A similar structure is also observed in the case of microfibers in our optical microscope.

**Figure 12:**
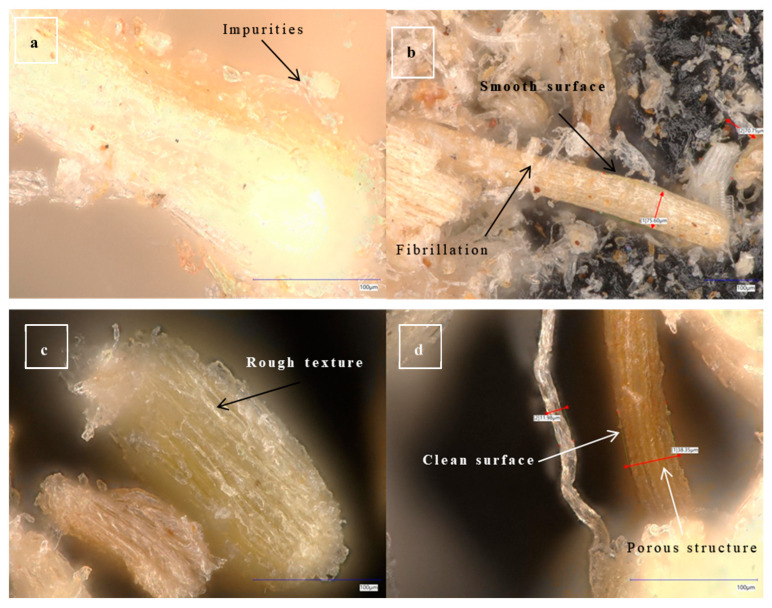
Optical microscopy images (**a**,**b**) untreated microfiber; (**c**,**d**) treated micro-fiber.

Figure 12 depicts the surface characteristics of untreated and alkali-treated date palm microfibers, as analyzed through optical microscopy. Untreated fibers displayed a smooth and compact surface morphology, with small fibrils and protrusions [54]. There is a noticeable presence of waxes, oils, and other impurities, as well as amorphous substances, in the fiber surface which contribute to their inherent binding properties in the raw fiber. In contrast, alkali-treated fibers exhibited an increase in surface roughness, accompanied by the emergence of pores on the fiber surface and the removal of small fibrils from the surface [55]. These surface modifications are attributed to the removal of waxes, oils, and impurities from the primary wall of the untreated fibers during the alkali treatment process.

### 4.6. Mechanical Properties Characterizations

#### 4.6.1. Tensile Properties

**Figure 13:**
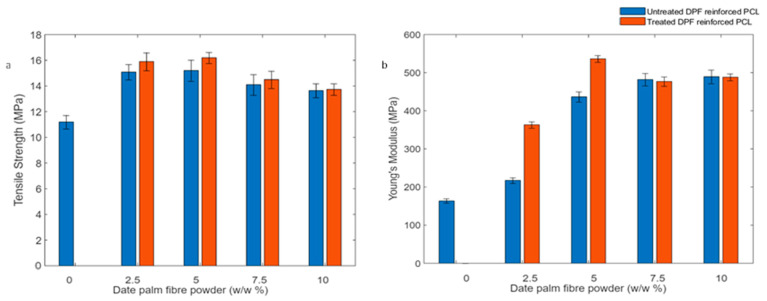
(**a**) Tensile strength and (**b**) Young’s Modulus of the untreated and treated date palm–PCL biocomposites.

The tensile strength of date palm microfiber-reinforced composites (DPMFC) is shown to improve with increasing microfiber content up to 5 wt.% with both untreated and alkali-treated fibers. In comparison to the neat PCL, the 5 wt.% MFC showed a 20% in tensile strength and a 164% increase in Young’s Modulus, as shown in Figure 13. This can be attributed to better adhesion between the natural microfibers and the polymer matrix at this optimal concentration, facilitating more effective stress transfer. However, beyond this point, the fibers tend to agglomerate, leading to imperfections in filler dispersion and, thus, a decline in tensile strength.

This trend is consistent with other natural fiber composites, where agglomeration at higher fiber loadings negatively impacts mechanical properties, as seen in studies involving henequen and other natural microfibers [29,56,57]. Increases in tensile modulus with higher microfiber content are also observed, though this improvement typically results in a more brittle composite, which aligns with findings on bacterial cellulose and PCL composite [58]. As the modulus increases, the composite becomes stiffer and less ductile, reducing the elongation at break. This is commonly attributed to the restriction of polymer chain mobility by the fibers, leading to decreased deformability, a trend observed in composites reinforced with microcrystalline cellulose (MCC) and other micro-size fillers [59].

For 5 wt.% DPMFC, the treated ones show a 10% increase in tensile strength and a 14.8% increase in Young’s Modulus in comparison to untreated composite samples. The tensile strength of date palm MFC is enhanced after alkali treatment because this process removes amorphous substances like lignin, hemicellulose, pectin, and other impurities, as evidenced by our FTIR study. The removal of these components makes the fiber surface rougher, as reported in our optical microscopy results, thereby improving the adhesion between the fiber and the matrix. Moreover, the increase in crystallinity of semi-crystalline polymers like PCL upon fiber reinforcement is also reported in our DSC results. The highest tensile strength and Young’s Modulus achieved with PCL reinforced by alkali-treated date palm microfibers are comparable to those of other filler-reinforced biocomposites, as summarized in Table 1, demonstrating their potential for application in packaging materials.

#### 4.6.2. Flexural Properties

**Figure 14:**
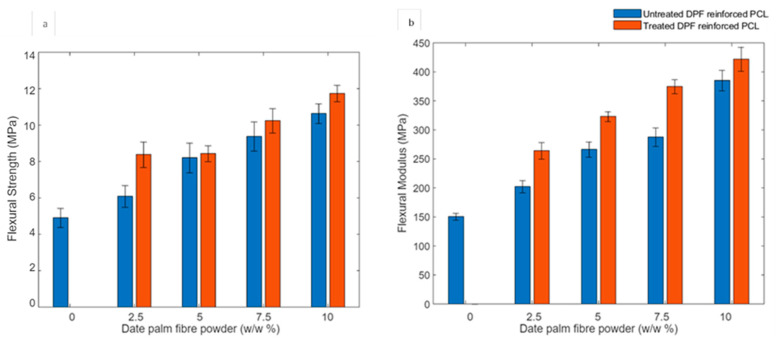
(**a**) Flexural strength and (**b**) flexural modulus of the untreated and treated date palm microfiber–PCL biocomposites.

The flexural behavior of polycaprolactone (PCL) and its composites reinforced with various percentages of microfibers was evaluated through bending tests at room temperature. As depicted in Figure 14, the flexural modulus increased substantially with the addition of microfiber.

Neat PCL exhibited a flexural modulus of 150 MPa, while composites containing 10% microfibers reached 375 MPa. The improvement in flexural properties was mainly influenced by microfiber content and the stiffness of the microfiber, with fiber–matrix interactions playing a lesser role. In the study conducted by Alemán-Domínguez et al., the addition of microcrystalline cellulose (MCC) to polycaprolactone (PCL) composites resulted in a 19% increase in flexural modulus with 2% *w*/*w* MCC and a 25% increase when the MCC content was raised to 5% *w*/*w* [59]. In contrast, our findings demonstrate significantly higher improvements, with the flexural modulus increasing by 66% and 120% for 2.5% *w*/*w* and 5% *w*/*w* MCC, respectively. The enhanced flexural strength of alkali-treated microfibers may be attributed to improved fiber–matrix adhesion due to fibrillation.

**Figure 15:**
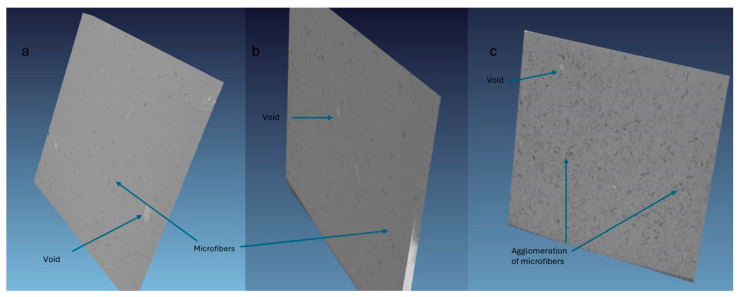
X-ray μCT micrographs of (**a**) 2.5 wt.%, (**b**) 5 wt.%, and (**c**) 10 wt.% date palm microfiber-reinforced PCL composites.

X-ray computed micro-tomography (μ-CT) scans of date palm microfiber-reinforced PCL composites reveal the fiber distribution at varying microfiber concentrations of 2.5 wt.%, 5 wt.%, and 10 wt.% as shown in Figure 15. The results indicate uniform fiber dispersion at 5 wt.%, whereas fiber agglomeration is observed at 10 wt.%. At 2.5 wt.%, the presence of noticeable porosity within the composite is evident. The increased modulus of the microfibers contributes to enhanced flexural strength and stiffness at higher microfiber concentrations.

### 4.7. Moisture Absorption Behavior

**Figure 16:**
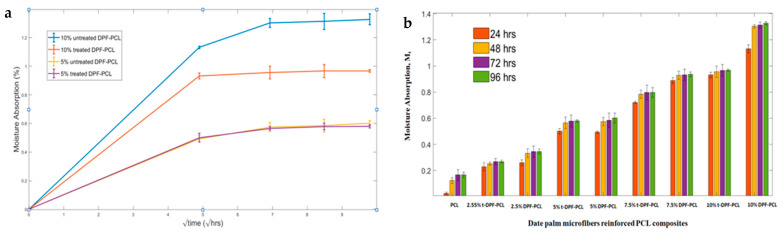
Moisture absorption curves (**a**) for 10% and 5% with respect to time; (**b**) moisture absorption of all samples.

The water absorption behavior of date palm microfiber (DPMF)-reinforced polycaprolactone (PCL) composites follows typical Fickian diffusion, as shown in Figure 16a, where water absorption increases rapidly in the early stages and then plateaus. The porous structure of natural fibers, along with capillary action, facilitates water transport into gaps between the matrix and fibers. With higher fiber loadings, water absorption increases due to the hydrophilic nature of natural fibers, which contain cellulose, hemicellulose, and lignin. These biopolymers possess polar hydroxyl groups that form hydrogen bonds with water, making composites with higher fiber content more susceptible to water uptake. The addition of fibers shifts the composite from hydrophobic to hydrophilic, which explains the observed increase in water absorption as fiber loading increases.

Alkali treatment of the fibers reduces water absorption at higher loadings. The change in the moisture absorption is not significant for 5% DPMF for both treated and untreated. However, there is a significant change in behavior for 10% of treated DPMF and untreated DPMF composites. This treatment removes hemicelluloses, and reduces the fiber’s ability to absorb more moisture [64]. Also, the removal of more hydroxyl groups decreases the fiber’s affinity for water [31]. The possible explanation for no change in the behavior for 5% is that for microfibers the treatment causes fibrillation and removed hydrophobic waxes, increasing the fiber’s surface area and making it more receptive to moisture. Thus, overall, there is no change in the moisture percentage for treated and untreated 5% DPMF composites. Other chemical or physical treatments are also necessary to increase the purity of cellulosic fiber and control the hydrophilic behavior. The results are in agreement with Benaniba and colleagues’ report that the weak interfacial adhesion between the untreated fibers and the matrix paves the way for water molecules to slip through more easily, leading to a significant spike in water absorption [65].

### 4.8. Water Contact Angle Analysis

Water contact angle measurements are widely used to assess the hydrophilic or hydrophobic nature of polymer surfaces, providing insight into material wettability. As shown in Table 2, the water contact angle for neat PCL samples was measured at 94.91°. The incorporation of DPF into the PCL matrix reduced the water contact angles proportionally to the fiber content, and a similar trend is also revealed in the incorporation of microcrystalline cellulose in the PCL matrix [59]. This reduction is attributed to the hydrophilic nature of the DPF and its effect on increasing surface roughness. Among the biocomposites investigated, the lowest water contact angle was observed for the 10% DPF-PCL composite, which contained the highest fiber content. The surface energy of pristine PCL is reported to range between 35 and 50 mN/m, depending on the method utilized for sample preparation [66]. The addition of hydrophilic fibers resulted in an increase in surface energy, indicative of enhanced hydrophilic behavior. At a low fiber loading of 2.5 wt.%, alkali-treated microfiber composites exhibited relatively lower surface energy. However, as the fiber concentration increased to 5, 7.5, and 10 wt.%, the variation in surface energy between the composites became less pronounced.

A comparison of untreated and alkali-treated DPF-PCL composites showed that the 2.5% DPF composite treated with 5% alkali solution exhibited the highest water contact angle (91.09°), closely approximating the value for neat PCL, while the untreated composite with the same filler content exhibited a lower angle of 82.13°. The increase in the contact angle for the alkali-treated DPF composites is attributed to enhanced fiber surface roughness following alkali treatment; the removal of amorphous components such as lignin, hemicelluloses, and wax; and the reaction of cellulose hydroxyl groups with NaOH, which lowers the fiber’s capacity to absorb water [67]. The findings reported by Royan and colleagues are in close agreement with our study on alkali-treated microfiber-based biocomposites [68]. Similar to their observation that alkali treatment enhances the hydrophobicity of rice husk composites by the removal of lignin, showing an increase in contact angle for the alkali-treated ones (93.5–100°) as compared to the untreated ones (89.3°), demonstrating improved water resistance and hydrophobic behavior. During alkali treatment, the amorphous components of date palm microfibers, including hemicellulose, lignin, and wax, were eliminated. This process reduced the number of hydroxyl groups available on the fiber surface [69].

### 4.9. Differential Scanning Calorimetry (DSC) Analysis Results

Differential scanning calorimetry (DSC) provides insights into the thermal behavior, crystallinity, and strength of polymers and polymeric composites [70]. By analyzing heat flow during melting and cooling, DSC determines the degree of crystallinity, which directly influences mechanical properties such as tensile strength, stiffness, and thermal resistance [71]. Higher crystallinity results in stronger intermolecular bonding, enhancing strength and reducing permeability, which is critical for packaging applications like food containers and films.

As indicated in Table 3, the inclusion of DPF microfibers into the composite slightly enhanced the χ _PCL_, likely due to the nucleating effect of the DPF microfibers on PCL crystallization [72]

The decrease in crystallinity of 10% treated DPF-PCL compared to 5% treated DPF-PCL can be explained by the balance between nucleation and polymer chain mobility. At 5% microfiber content, the fibers act as nucleating agents, providing crystallization points that enhance the regular arrangement of polymer chains, leading to smaller, more perfect crystals and increased crystallinity. However, at 10% microfiber content, the higher filler concentration restricts the mobility of polymer chains and disrupts the crystal lattice structure, reducing crystallinity. Additionally, excessive microfiber loading can lead to agglomeration, decreasing the effective nucleation sites. This trend aligns with studies, such as those by Tomec [73] et al., showing similar behavior in MCC-reinforced biocomposites.

**Figure 17:**
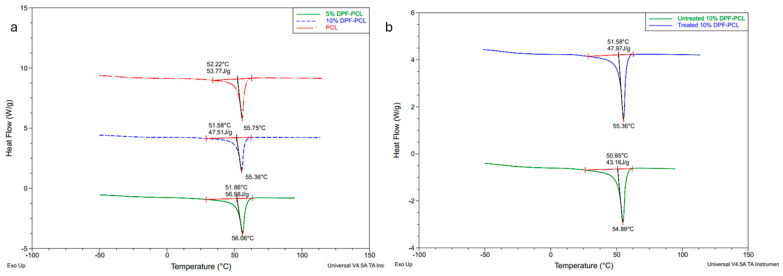
DSC heating curves (**a**) for neat PCL with 5 and 10% DPF-PCL composite samples (**b**) for treated and untreated DPMF composite samples.

The NaOH treatment, as depicted in Figure 17, facilitated the removal of amorphous components like hemicellulose and pectin from the DPF surface, which is corroborated by FTIR analysis. This treatment resulted in an increase in the crystalline regions of the microfibers, as reported in the XRD results, thereby enhancing the χ _PCL_ of the composites. The melting temperature, T_m_, is also increased in the case of treated DPF composites. The crystallinity of the matrix exhibits a trend, increasing from 38.6% for neat PCL to 43.1% for the 5% DPF composite but decreasing slightly to 35.6% at 10% DPF loading. The crystallinity decreases for the 10% DPF-PCL composite compared to the neat PCL. At high concentrations of microfibers, the mobility of the molecular chains may be reduced due to the presence of the microfibers, which hinders crystal formation and lowers the overall effectiveness of the filler [74].

### 4.10. Dynamic Mechanical Analysis (DMA) Results

Dynamic Mechanical Analysis (DMA) is crucial for understanding the viscoelastic behavior of polymers and polymeric composites across a wide temperature range [75]. For packaging applications, DMA is particularly relevant because it helps evaluate how polymers will perform under varying environmental conditions, such as temperature fluctuations during transportation or storage.

**Figure 18:**
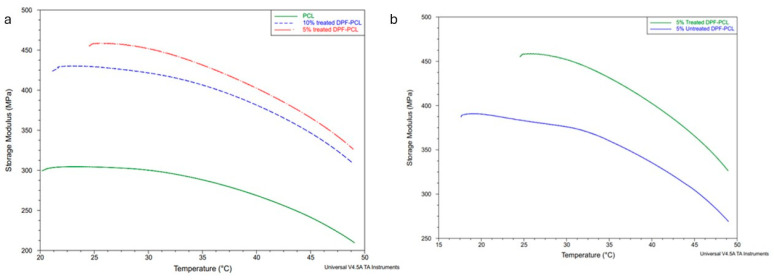
(**a**) Storage modulus curve for neat PCL and different DPMF composites; (**b**) storage modulus for treated and untreated DPMF composites.

In the DMA, results depicted in Figure 18 illustrate that the inclusion of date palm microfiber into the PCL matrix led to an increase in the storage modulus, with the magnitude of the increase being dependent on the fiber contents. Specifically, both 5% and 10% DPF-PCL samples exhibited an increase in storage modulus relative to the neat PCL matrix, reflecting an improvement in stiffness due to the incorporation of fibers [26]. This is consistent with previous findings where tensile modulus showed similar improvements with the addition of date palm microfibers to the PCL matrix. Notably, the 5% DPF-PCL samples demonstrated higher storage modulus across the entire temperature range when compared to the 10% DPF-PCL composites. This behavior may be attributed to the reduced degree of crystallinity observed in the 10% DPF-PCL samples, as indicated by the differential scanning calorimetry (DSC) analysis. The higher fiber content in composites can lead to fiber agglomeration and decreased fiber–matrix adhesion, which negatively impacts mechanical properties as evidenced by the lower storage modulus of 10% DPF-PCL samples.

Alkali-treated date palm microfiber composites exhibited higher storage moduli compared to untreated fiber composites, as depicted in Figure 18b. This enhancement in storage modulus is more pronounced in the PCL composites reinforced with 5% alkali-treated DPF. The improvement in storage modulus for alkali-treated fibers can be attributed to the removal of surface impurities and, as a result, a better dispersion of fibers within the PCL matrix. The improvement in fiber–matrix adhesion resulting from alkali treatment is further supported by DSC results, which showed a slight increase in the degree of crystallinity for alkali-treated DPF-PCL composites compared to their untreated counterparts.

### 4.11. Rheological Behavior

Understanding the rheological behavior of polymers is crucial for evaluating the impact of fillers on their flow properties and optimizing processes like injection molding [76]. Fillers alter a polymer’s viscosity, elasticity, and shear response, influencing how the material flows under high pressure and shear conditions, which is critical for uniform mold filling and defect-free products.

**Figure 19:**
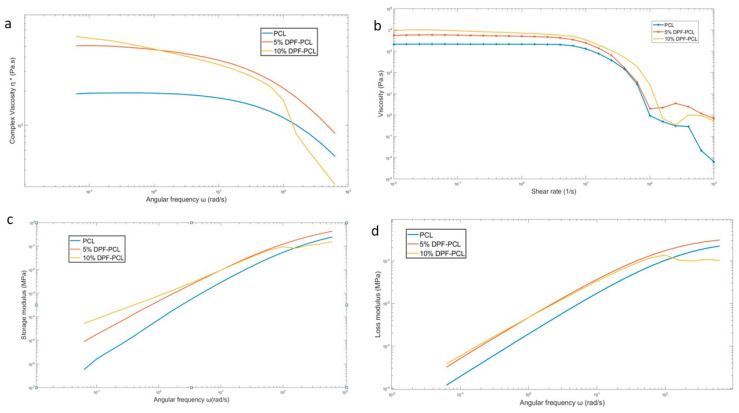
(**a**) Viscosity with respect to frequency; (**b**) viscosity with respect to shear rate; (**c**) storage modulus; (**d**) loss modulus with respect to frequency.

Figure 19a presents the complex viscosity (η*) as a function of angular frequency (ω) for neat PCL, 5% DPF-PCL, and 10% DPF-PCL. The addition of DPF significantly increased the melt viscosity of the composites compared to the neat PCL, with a more pronounced effect observed at higher filler content. This increase in viscosity is indicative of the hindrance to polymer chain mobility caused by the presence of fibers, which introduces additional resistance to flow [77]. The non-Newtonian behavior is more noticeable for samples with fillers compared with just neat PCL samples. The observed non-Newtonian behavior at low frequencies is primarily due to the strong fiber–fiber interactions, resulting in a network structure that imparts enhanced rigidity and resistance to deformation. The incorporation of DPF into the PCL matrix not only disrupts the polymer flow but also induces hydrodynamic effects, as seen with other filler materials. As the concentration of fibers increases, these effects become more significant, impacting both the elasticity and viscosity across the entire frequency range [78].

At low frequencies, the biocomposites exhibited a solid-like behavior, suggesting the formation of a percolated fiber network within the polymer matrix and network structure results in a yield stress phenomenon. The effect of fiber content on the rheological properties was more pronounced at low frequencies, where filler–filler interactions dominate the behavior of the composite [79]. At low frequencies, the increased filler content led to a significant enhancement in the storage modulus, while at high frequencies, the storage modulus tended to be similar to that of the neat matrix. At higher frequencies, the fiber network is broken, and the polymer controls the overall behavior of the composite [80].

**Figure 20:**
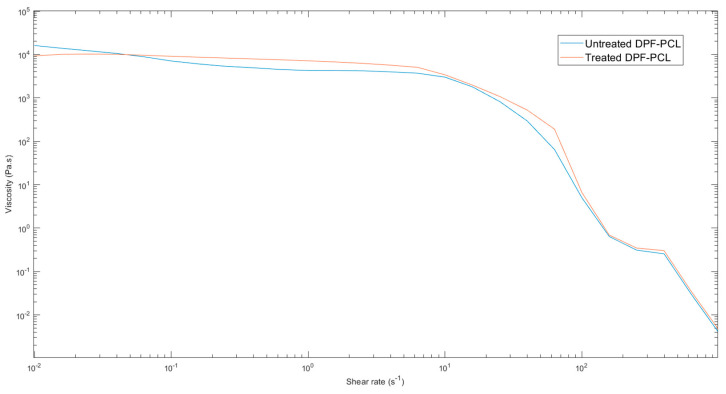
Curves of viscosity versus shear rate for alkali-treated DPF and untreated DPF.

The alkali treatment of date palm microfibers may lead to various interactions, such as hydrodynamic interactions and fiber–matrix interactions, all of which can influence the viscoelastic properties of molten biocomposites materials [78]. The viscosity of alkali-treated DPF-reinforced PCL composites is slightly higher than that of untreated composites, as shown in Figure 20, which may possibly be attributed to structural modifications on the surface of the date palm microfibers caused by alkali treatment. Both the treated and untreated composites show pseudoplastic behavior, with higher viscosity at low shear rates due to high fiber–fiber interactions and reduced viscosity at higher shear rates as fibers align, and the effect of treatment is not considerable at high shear rates [81].

### 4.12. TGA of Biocomposites

**Figure 21:**
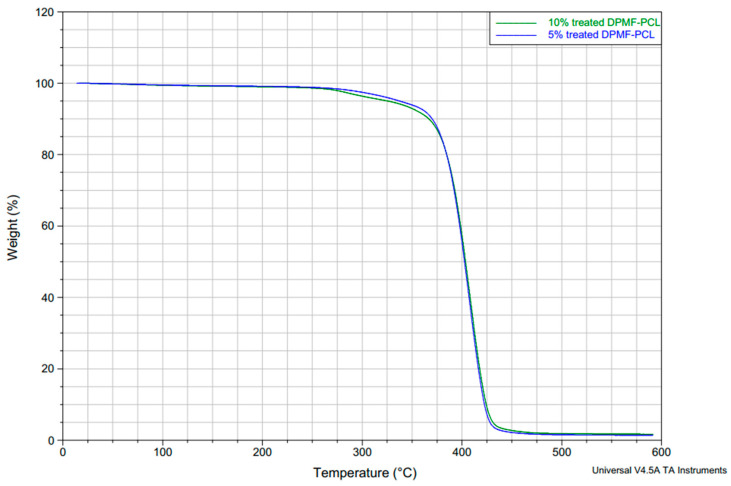
TGA of 5% and 10% treated DPMF-PCL biocomposites.

Thermogravimetric Analysis (TGA) curves presented in Figure 21 show the weight loss (%) as a function of temperature for two composites: 5% treated DPMF-reinforced PCL and 10% treated DPMF-reinforced PCL. From the TGA curves you provided, the onset temperature for decomposition of the 10% treated DPF-PCL composite does appear to be slightly lower than that of the 5% treated DPF-PCL composite. This can be interpreted as the 10% fiber-reinforced composite having a marginally reduced thermal stability compared to the 5% composite. The lower thermal stability observed in the 10% fiber composite compared to the 5% composite can be attributed to higher fiber agglomeration and uneven dispersion within the polymer matrix, and this is in agreement with the X-ray μCT micrographs illustrated in Figure 15. Natural fibers, such as date palm fibers, are mainly made up of cellulose and hemicellulose, which break down at much lower temperatures than most polymer matrices [82]. As a result, they decompose earlier when used in composite materials, and increasing the fiber content amplifies this effect by adding more thermally unstable components [83].

### 4.13. Surface Morphology Analysis

**Figure 22:**
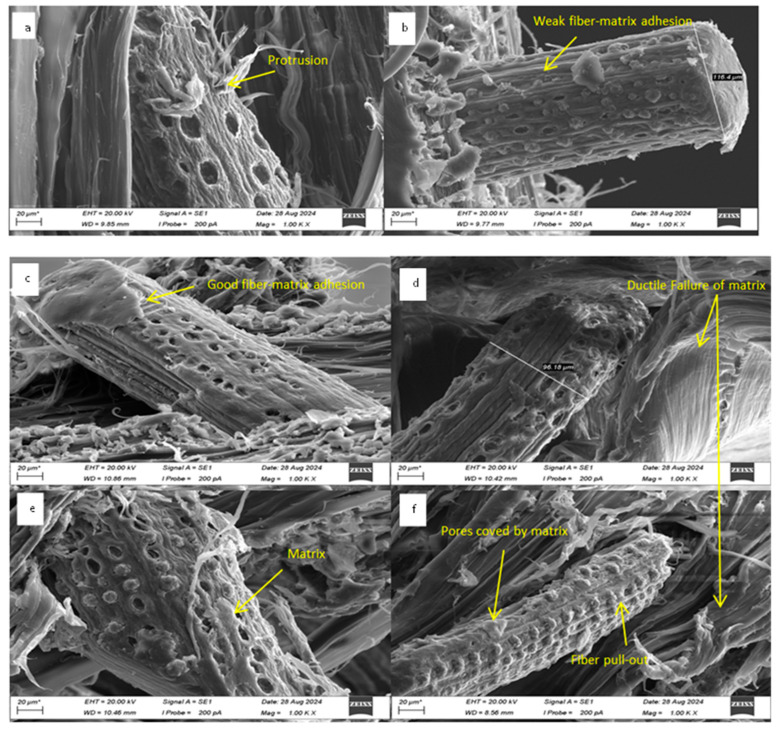
SEM images of fractured region; (**a**,**b**) untreated date palm fiber composites; (**c**–**f**) treated date palm fiber composites.

Scanning electron microscopy (SEM) analysis of fractured surfaces of tensile tested samples reveals enhanced interaction between date palm microfibers (DPMF) and the polycaprolactone (PCL) matrix following alkali treatment. The surface of the untreated DPF-PCL shows a cleaner and smoother surface compared to the treated DPF-PCL, indicating a weaker adhesion between DPF and PCL [84]. The surface morphology of alkali-treated fibers exhibited significant differences from untreated fibers, particularly in terms of smoothness and roughness. SEM micrographs (Figure 22a) demonstrate the presence of surface impurities on untreated fibers, whereas micrographs of fibers treated with 5% NaOH for 3 h reveal the removal of these impurities. The treated fibers exhibit surface scrapes, which correlate with the removal of hemicellulose and lignin, as confirmed by Fourier-transform infrared (FTIR) spectroscopy.

In NaOH-treated composites, improved fiber–matrix adhesion is evident, showing a significant increase in the tensile strength of 5% concentrated treated composites, with a substantial amount of PCL adhering to the fiber surface compared to untreated composites. This enhancement is attributed to the fibrillation induced by alkali treatment, which increases the available surface area for matrix interaction and improves wettability [85]. These findings demonstrate that alkali treatment significantly improves the interfacial bonding between DPF and the PCL matrix. The analysis also highlights increased fiber pull-out in composites containing 5% NaOH-treated fibers compared to untreated DPF-PCL composites, underscoring the enhanced load-bearing capacity of the treated fibers due to improved wettability [86]. The SEM images of alkali-treated DPF-PCL composites further validate improved fiber–matrix bonding, as shown in Figure 22c, attributed to the removal of non-cellulosic components and increased surface roughness caused by chemical treatment.

## 5. Conclusions

This study underscores the potential of date palm agricultural residues, particularly date palm microfibers (DPMFs), as sustainable reinforcements in biopolymer composites. Through systematic evaluation, it has been demonstrated that alkali-treated DPMF effectively enhances the mechanical, thermal, and physicochemical properties of polycaprolactone (PCL) biocomposites. Alkali treatment significantly improves the surface characteristics of DPMF, facilitating better interfacial bonding with the PCL matrix, as confirmed by spectroscopic, thermal, and microscopy analyses. Moreover, the effect of alkali treatment on the crystallinity and the crystal size of the date palm microfiber has been investigated and reported. The resulting biocomposites exhibit notable enhancements in tensile strength, water-repellence behavior, thermal stability, and storage modulus, with the optimal performance observed at 5 wt.% micro-fiber loading. Moreover, the findings emphasize the environmental and economic benefits of utilizing date palm residues for key applications, which are abundantly available but underutilized. The study contributes to the growing body of research on agricultural waste valorization, presenting a pathway to mitigate environmental pollution while advancing sustainable packaging applications. The dry blending, in combination with the compression molding approach outlined in this study, offers an energy-efficient and scalable method for biocomposite fabrication, highlighting its feasibility for industrial implementation. Overall, alkali-treated DPMF-reinforced PCL biocomposites demonstrate a significant promise for semi-structural and rigid packaging applications as evidenced by the obtained results.

## Figures and Tables

**Table 1:** Comparison of tensile strength and modulus of various bio-polymers and fillers for packaging application.

Polymer	Fillers	Tensile Strength	Young’s Modulus	Reference
Poly(butylene succinate-co-butylene adipate) (PBSA) and poly(butylene adipate-co-terephthalate) (PBAT)	Walnut shell powder	13 MPa	470 MPa	[60]
Poly(butylene succinate-co-butylene adipate) (PBSA)	Waste almond shell powder	14 MPa	1100 MPa	[61]
Polylactic acid (PLA)	Chitosan	35 MPa	970 MPa	[62]
Starch	Calcium carbonate	13.6 MPa	-	[63]
PCL	5% treated DPMF	16.5 MPa	550 MPa	(From this study)

**Table 2:** Contact angle measurement of untreated and alkali-treated biocomposites representing its calculated average and standard deviation.

Sample	Image	Water Contact Angle	Surface Energy (mN/m)	Sample	Image	Water Contact Angle	Surface Energy (mN/m)
100% PCL	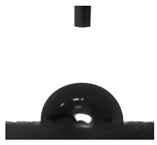	94.91 ± 2.6	55.96 ± 1.5				
2.5% DPF-PCL	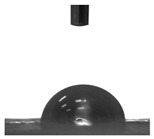	82.13 ± 1.8	62.04 ± 2.1	2.5% Alkali treated DPF-PCL	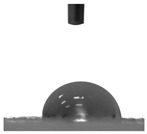	91.09 ± 1.3	56.92 ± 0.72
5% DPF-PCL	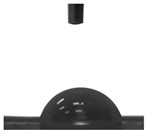	73.04 ± 1.2	82.88 ± 3.13	5% treated DPF-PCL	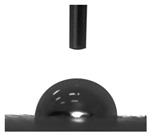	88.42 ± 0.56	86.81 ± 1.43
7.5% DPF-PCL	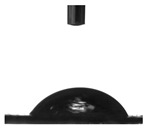	60.24 ± 1.34	82.44 ± 2.74	7.5% treated DPF-PCL	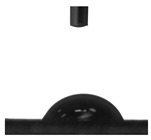	66.96 ± 0.83	87.66 ± 2.04
10% DPF-PCL	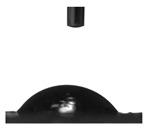	58.80 ± 1.40	89.53 ± 1.30	10% treated DPF-PCL	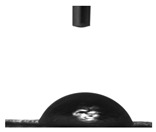	63.79 ± 0.89	89.63 ± 1.21

**Table 3:** Degree of crystallinity at different concentrations of treated and untreated samples.

Specimens	Degree of Crystallinity (%)
PCL	38.6
5% treated DPF-PCL	43.1
10% treated DPF-PCL	35.6
10% untreated DPF-PCL	34.5

## Data Availability

Data are contained within the article.

## References

[1] Rambabu K., Bharath G., Avornyo A., Thanigaivelan A., Hai A., Banat F. (2023). Valorization of date palm leaves for adsorptive remediation of 2,4-dichlorophenoxyacetic acid herbicide polluted agricultural runoff. Environ. Pollut..

[2] Belgacem C., Serra-Parareda F., Tarrés Q., Mutjé P., Delgado-Aguilar M., Boufi S. (2021). The Integral Utilization of Date Palm Waste to Produce Plastic Composites. Polymers.

[3] Sadh P.K., Duhan S., Duhan J.S. (2018). Agro-industrial wastes and their utilization using solid state fermentation: A review. Bioresour. Bioprocess..

[4] Berthet M.A., Angellier-Coussy H., Chea V., Guillard V., Gastaldi E., Gontard N. (2015). Sustainable food packaging: Valorising wheat straw fibres for tuning PHBV-based composites properties. Compos. Part A Appl. Sci. Manuf..

[5] Bangar S.P., Whiteside W.S., Kajla P., Tavassoli M. (2023). Value addition of rice straw cellulose fibers as a reinforcer in packaging applications. Int. J. Biol. Macromol..

[6] Al-Awa Z.F.A., Sangor F.I.M.S., Babili S.B., Saud A., Saleem H., Zaidi S.J. (2023). Effect of Leaf Powdering Technique on the Characteristics of Date Palm-Derived Cellulose. ACS Omega.

[7] Fethiza Tedjani C., Ben Mya O., Rebiai A. (2020). Isolation and characterization of cellulose from date palm tree spathe sheath. Sustain. Chem. Pharm..

[8] Galiwango E., Abdel Rahman N.S., Al-Marzouqi A.H., Abu-Omar M.M., Khaleel A.A. (2019). Isolation and characterization of cellulose and α-cellulose from date palm biomass waste. Heliyon.

[9] Alothman O.Y., Shaikh H.M., Alshammari B.A., Jawaid M. (2022). Structural, Morphological and Thermal Properties of Nano Filler Produced from Date Palm-Based Micro Fibers (*Phoenix dactylifera* L.). J. Polym. Environ..

[10] Amara C., El Mahdi A., Medimagh R., Khwaldia K. (2021). Nanocellulose-based composites for packaging applications. Curr. Opin. Green Sustain. Chem..

[11] Lima E.M.B., Middea A., Neumann R., Thiré R.M.d.S.M., Pereira J.F., de Freitas S.C., Penteado M.S., Lima A.M., Minguita A.P.d.S., Mattos M.d.C. (2021). Biocomposites of PLA and Mango Seed Waste: Potential Material for Food Packaging and a Technological Alternative to Reduce Environmental Impact. Starch-Stärke.

[12] Elhussieny A., Faisal M., D’Angelo G., Aboulkhair N.T., Everitt N.M., Fahim I.S. (2020). Valorisation of shrimp and rice straw waste into food packaging applications. Ain Shams Eng. J..

[13] Thivya P., Bhosale Y., Anandakumar S., Hema V., Sinija V. (2021). Exploring the effective utilization of shallot stalk waste and tamarind seed for packaging film preparation. Waste Biomass Valorization.

[14] Bilo F., Pandini S., Sartore L., Depero L.E., Gargiulo G., Bonassi A., Federici S., Bontempi E. (2018). A sustainable bioplastic obtained from rice straw. J. Clean. Prod..

[15] Boughezal A., Ben Mya O., Lanez T., Fethiza Tedjani C. (2023). Extraction of pure cellulose from palm residues using alkaline treatment method and its performance in PVC polymer matrix composite. Biomass Convers. Biorefinery.

[16] Saputro A., Verawati I., Ramahdita G., Chalid M. (2017). Preparation of micro-fibrillated cellulose based on sugar palm ijuk (Arenga pinnata) fibres through partial acid hydrolysis. IOP Conf. Ser. Mater. Sci. Eng..

[17] Hachaichi A., Kouini B., Kian L.K., Asim M., Jawaid M. (2021). Extraction and Characterization of Microcrystalline Cellulose from Date Palm Fibers using Successive Chemical Treatments. J. Polym. Environ..

[18] Khatun M.A., Sultana S., Islam Z., Kabir M.S., Hossain M.S., Nur H.P., Chowdhury A.M.S. (2023). Extraction of crystalline nanocellulose (CNC) from date palm mat fibers and its application in the production of nanocomposites with polyvinyl alcohol and polyvinylpyrrolidone blended films. Results Eng..

[19] Bangar S.P., Kajla P., Ghosh T. (2023). Valorization of wheat straw in food packaging: A source of cellulose. Int. J. Biol. Macromol..

[20] Harini K., Chandra Mohan C. (2020). Isolation and characterization of micro and nanocrystalline cellulose fibers from the walnut shell, corncob and sugarcane bagasse. Int. J. Biol. Macromol..

[21] Raza M., Mustafa J., Al-Marzouqi A.H., Abu-Jdayil B. (2024). Isolation and characterization of cellulose from date palm waste using rejected brine solution. Int. J. Thermofluids.

[22] Phanthong P., Reubroycharoen P., Hao X., Xu G., Abudula A., Guan G. (2018). Nanocellulose: Extraction and application. Carbon Resour. Convers..

[23] Nassar M.M.A., Alzebdeh K.I., Al-Hinai N., Safy M.A. (2024). Enhancing mechanical performance of polypropylene bio-based composites using chemically treated date palm filler. Ind. Crops Prod..

[24] Mousa N., Galiwango E., Haris S., Al-Marzouqi A.H., Abu-Jdayil B., Caires Y.L. (2022). A New Green Composite Based on Plasticized Polylactic Acid Mixed with Date Palm Waste for Single-Use Plastics Applications. Polymers.

[25] Pérez-Fonseca A.A., Martín Del Campo A.S., Robledo-Ortíz J.R., González-López M.E. (2022). Compatibilization strategies for PLA biocomposites: A comparative study between extrusion-injection and dry blending-compression molding. Compos. Interfaces.

[26] Saifullah A., Chacko N.G., Dhakal H.N., Khan S.H., Sarker F., Zhang Z. (2024). Valorisation of Agricultural Residue Bio-Mass Date Palm Fibre in Dry-Blended Polycaprolactone (PCL) Bio-Composites for Sustainable Packaging Applications. Waste Biomass Valorization.

[27] Carotenuto M.R., Cavallaro G., Chinnici I., Lazzara G., Milioto S. (2024). Hybrid Green Materials Obtained by PCL Melt Blending with Diatomaceous Earth. Molecules.

[28] Dhakal H.N., Khan S.H., Alnaser I.A., Karim M.R., Saifullah A., Zhang Z. (2024). Potential of Date Palm Fibers (DPFs) as a Sustainable Reinforcement for Bio- Composites and its Property Enhancement for Key Applications: A Review. Macromol. Mater. Eng..

[29] Dhakal H., Bourmaud A., Berzin F., Almansour F., Zhang Z., Shah D.U., Beaugrand J. (2018). Mechanical properties of leaf sheath date palm fibre waste biomass reinforced polycaprolactone (PCL) biocomposites. Ind. Crops Prod..

[30] Bekele A.E., Lemu H.G., Jiru M.G. (2023). Study of the Effects of Alkali Treatment and Fiber Orientation on Mechanical Properties of Enset/Sisal Polymer Hybrid Composite. J. Compos. Sci..

[31] Kamaruddin Z.H., Jumaidin R., Ilyas R.A., Selamat M.Z., Alamjuri R.H., Yusof F.A.M. (2022). Influence of Alkali Treatment on the Mechanical, Thermal, Water Absorption, and Biodegradation Properties of Cymbopogan citratus Fiber-Reinforced, Thermoplastic Cassava Starch–Palm Wax Composites. Polymers.

[32] Vijay R., Vinod A., Lenin Singaravelu D., Sanjay M.R., Siengchin S. (2021). Characterization of chemical treated and untreated natural fibers from Pennisetum orientale grass- A potential reinforcement for lightweight polymeric applications. Int. J. Lightweight Mater. Manuf..

[33] Jing L., Jiang Y., Li L., Zhang T. (2023). Influence of Alkali Treatment on Microstructure Transformation and Mechanical Properties of Palm-Fiber Cell Wall. J. Nat. Fibers.

[34] Amior A., Satha H., Laoutid F., Toncheva A., Dubois P. (2023). Natural Cellulose from Ziziphus jujuba Fibers: Extraction and Characterization. Materials.

[35] (2015). Standard Test Method for Tensile Properties of Plastics.

[36] (2019). Standard Test Methods for Flexural Properties of Unreinforced and Reinforced Plastics and Electrical Insulating Materials 1.

[37] Wong E.H., Rajoo R. (2003). Moisture absorption and diffusion characterisation of packaging materials––Advanced treatment. Microelectron. Reliab..

[38] Mucha M., Tylman M., Mucha J. (2015). Crystallization kinetics of polycaprolactone in nanocomposites. Polimery.

[39] Reddy K.O., Reddy K.R.N., Zhang J., Zhang J., Varada Rajulu A. (2013). Effect of Alkali Treatment on the Properties of Century Fiber. J. Nat. Fibers.

[40] Alawar A., Hamed A.M., Al-Kaabi K. (2009). Characterization of treated date palm tree fiber as composite reinforcement. Compos. Part B Eng..

[41] Ikramullah, Rizal S., Thalib S., Huzni S. (2018). Hemicellulose and lignin removal on typha fiber by alkali treatment. IOP Conf. Ser. Mater. Sci. Eng..

[42] Bellel N., Bellel N. (2023). Sustainable heat insulation composites based on Portland cement reinforced with date palm fibers. J. Eng. Fibers Fabr..

[43] Sheeba K.R.J., Alagarasan J.K., Dharmaraja J., Kavitha S.A., Shobana S., Arvindnarayan S., Vadivel M., Lee M., Retnam K.P. (2023). Physico–chemical and extraction properties on alkali–treated Acacia pennata fiber. Environ. Res..

[44] Oudiani A.E., Chaabouni Y., Msahli S., Sakli F. (2011). Crystal transition from cellulose I to cellulose II in NaOH treated *Agave americana* L. fibre. Carbohydr. Polym..

[45] Shahril S.M., Ridzuan M.J.M., Majid M.S.A., Bariah A.M.N., Rahman M.T.A., Narayanasamy P. (2022). Alkali treatment influence on cellulosic fiber from Furcraea foetida leaves as potential reinforcement of polymeric composites. J. Mater. Res. Technol..

[46] ŞEnay R., ÖZdemİR H., Seki Y., Keskin Ö., Dalmis R., Koktas S., ErdoĞAn Ü. (2022). Comparison of alkali concentration for obtaining fine Musa Sapientum (banana) fibers to enhance potential applications. Tekst. Konfeksiyon.

[47] Alsaeed T., Yousif B.F., Ku H. (2013). The potential of using date palm fibres as reinforcement for polymeric composites. Mater. Des..

[48] Geremew A., De Winne P., Demissie T.A., De Backer H. (2024). Surface modification of bamboo fibers through alkaline treatment: Morphological and physical characterization for composite reinforcement. J. Eng. Fibers Fabr..

[49] Pitchayya Pillai G., Manimaran P., Vignesh V. (2021). Physico-chemical and Mechanical Properties of Alkali-Treated Red Banana Peduncle Fiber. J. Nat. Fibers.

[50] Ebissa D.T., Tesfaye T., Worku D., Wood D. (2022). Characterization and optimization of alkali-treated yushania alpina bamboo fiber properties: Case study of ethiopia species. SN Appl. Sci..

[51] García-Méndez R.F., Cortés-Martínez C.I., Carrillo J.G., Almendárez-Camarillo A. (2023). Investigation on Physicochemical, Tensile Test, and Thermal Properties of Alkali Treatment to A. Angustifolia Haw Fibers. J. Nat. Fibers.

[52] Song X., Huang C., Qin H., Guan W., Ye Y. (2022). Effects of alkali treatment on properties of willow bark fiber as potential fillers for polymer composites. J. Eng. Fibers Fabr..

[53] Melelli A., Arnould O., Beaugrand J., Bourmaud A. (2020). The Middle Lamella of Plant Fibers Used as Composite Reinforcement: Investigation by Atomic Force Microscopy. Molecules.

[54] Kandel K.P., Adhikari M., Kharel M., Aryal G.M., Pandeya S., Joshi M.K., Dahal B., Gautam B., Neupane B.B. (2022). Comparative study on material properties of wood-ash alkali and commercial alkali treated Sterculia fiber. Cellulose.

[55] Khan S.H., Rahman M.Z., Haque M.R., Hoque M.E., Khiari R., Jawaid M., Belgacem M.N. (2023). Characterization and Comparative Evaluation of Structural, Chemical, Thermal, Mechanical, and Morphological Properties of Plant Fibers. Annual Plant: Sources of Fibres, Nanocellulose and Cellulosic Derivatives: Processing, Properties and Applications.

[56] Kim J., Cho D. (2022). Effects of Alkali-Treatment and Feeding Route of Henequen Fiber on the Heat Deflection Temperature, Mechanical, and Impact Properties of Novel Henequen Fiber/Polyamide 6 Composites. J. Compos. Sci..

[57] Ng Y.R., Shahid S.N.A.M., Nordin N.I.A.A. (2018). The effect of alkali treatment on tensile properties of coir/polypropylene biocomposite. IOP Conf. Ser. Mater. Sci. Eng..

[58] Kotcharat P., Chuysinuan P., Thanyacharoen T., Techasakul S., Ummartyotin S. (2021). Development of bacterial cellulose and polycaprolactone (PCL) based composite for medical material. Sustain. Chem. Pharm..

[59] Alemán-Domínguez M.E., Giusto E., Ortega Z., Tamaddon M., Benítez A.N., Liu C. (2019). Three-dimensional printed polycaprolactone-microcrystalline cellulose scaffolds. J. Biomed. Mater. Res. B Appl. Biomater..

[60] McNeill D.C., Pal A.K., Mohanty A.K., Misra M. (2024). Injection molding of biodegradable polyester blends filled with mineral and sustainable fillers: Performance evaluation. J. Appl. Polym. Sci..

[61] Root K.P., Pal A.K., Pesaranhajiabbas E., Mohanty A.K., Misra M. (2023). Injection moulded composites from high biomass filled biodegradable plastic: Properties and performance evaluation for single-use applications. Compos. Part C Open Access.

[62] Singh A.A., Sharma S., Srivastava M., Majumdar A. (2020). Modulating the properties of polylactic acid for packaging applications using biobased plasticizers and naturally obtained fillers. Int. J. Biol. Macromol..

[63] Navasingh R.J.H., Gurunathan M.K., Nikolova M.P., Królczyk J.B. (2023). Sustainable Bioplastics for Food Packaging Produced from Renewable Natural Sources. Polymers.

[64] Mohammed M., Jawad A.J.a.M., Mohammed A.M., Oleiwi J.K., Adam T., Osman A.F., Dahham O.S., Betar B.O., Gopinath S.C.B., Jaafar M. (2023). Challenges and advancement in water absorption of natural fiber-reinforced polymer composites. Polymer Testing.

[65] Benaniba S., Djendel M., Kessal O., Abderraouf B.A., Boubaaya R., Dridi M., Driss Z. (2023). Evaluation of mechanical properties of biocomposites treated with date palm fiber. J. Eng. Fibers Fabr..

[66] Jirkovec R., Erben J., Sajdl P., Chaloupek J., Chvojka J. (2021). The effect of material and process parameters on the surface energy of polycaprolactone fibre layers. Mater. Des..

[67] Vandna, Yadav V.L. (2024). Influence of alkali treatment on coir-reinforced polyvinyl alcohol/polyethylene glycol blends. J. Plast. Film. Sheeting.

[68] Rajendran Royan N.R., Sulong A.B., Yuhana N.Y., Chen R.S., Ab Ghani M.H., Ahmad S. (2018). UV/O3 treatment as a surface modification of rice husk towards preparation of novel biocomposites. PLoS ONE.

[69] Ikramullah I. (2019). Tailoring the Effective Properties of Typha Fiber Reinforced Polymer Composite via Alkali Treatment. BioResources.

[70] Balani K., Verma V., Agarwal A., Narayan R. (2015). Biosurfaces: A Materials Science and Engineering Perspective.

[71] Kong Y., Hay J.N. (2002). The measurement of the crystallinity of polymers by DSC. Polymer.

[72] Yang J.-Y., Kim D.-K., Han W., Park J.-Y., Kim K.-W., Kim B.-J. (2022). Effect of Nucleating Agents Addition on Thermal and Mechanical Properties of Natural Fiber-Reinforced Polylactic Acid Composites. Polymers.

[73] Krapež Tomec D., Schöflinger M., Leßlhumer J., Gradišar Centa U., Žigon J., Kariž M. (2024). The Effects of Microcrystalline Cellulose Addition on the Properties of Wood–PLA Filaments for 3D Printing. Polymers.

[74] Tarani E., Arvanitidis I., Christofilos D., Bikiaris D.N., Chrissafis K., Vourlias G. (2023). Calculation of the degree of crystallinity of HDPE/GNPs nanocomposites by using various experimental techniques: A comparative study. J. Mater. Sci..

[75] Akil H., Zamri M.H., Hodzic A., Shanks R. (2014). 12—Performance of natural fiber composites under dynamic loading. Natural Fibre Composites.

[76] Andrzejewski J., Barczewski M., Szostak M. (2019). Injection Molding of Highly Filled Polypropylene-based Biocomposites. Buckwheat Husk and Wood Flour Filler: A Comparison of Agricultural and Wood Industry Waste Utilization. Polymers.

[77] Botta L., Titone V., Mistretta M.C., La Mantia F.P., Modica A., Bruno M., Sottile F., Lopresti F. (2021). PBAT Based Composites Reinforced with Microcrystalline Cellulose Obtained from Softwood Almond Shells. Polymers.

[78] Borchani K.E., Carrot C., Jaziri M. (2019). Rheological behavior of short Alfa fibers reinforced Mater-Bi^®^ biocomposites. Polymer Test..

[79] Madera-Santana T., Misra M., Drzal L., Robledo D., Freile-Pelegrin Y. (2009). Preparation and characterization of biodegradable agar/poly (butylene adipate-co-terephatalate) composites. Polym. Eng. Sci..

[80] Scaffaro R., Botta L., Ceraulo M., La Mantia F. (2011). Effect of kind and content of organo-modified clay on properties of PET nanocomposites. J. Appl. Polym. Sci..

[81] Mukherjee M., Kumar S., Bose S., Das C.K., Kharitonov A.P. (2008). Study on the Mechanical, Rheological, and Morphological Properties of Short Kevlar™ Fiber/s-PS Composites. Polym.-Plast. Technol. Eng..

[82] Neto J.S.S., de Queiroz H.F.M., Aguiar R.A.A., Banea M.D. (2021). A Review on the Thermal Characterisation of Natural and Hybrid Fiber Composites. Polymers.

[83] Dorez G., Ferry L., Sonnier R., Taguet A., Lopez-Cuesta J.M. (2014). Effect of cellulose, hemicellulose and lignin contents on pyrolysis and combustion of natural fibers. J. Anal. Appl. Pyrolysis.

[84] Aydın M., Tozlu H., Kemaloglu S., Aytac A., Ozkoc G. (2011). Effects of Alkali Treatment on the Properties of Short Flax Fiber–Poly(Lactic Acid) Eco-Composites. J. Polym. Environ..

[85] Abdal-hay A., Suardana N.P.G., Jung D.Y., Choi K.-S., Lim J.K. (2012). Effect of diameters and alkali treatment on the tensile properties of date palm fiber reinforced epoxy composites. Int. J. Precis. Eng. Manuf..

[86] Singh J.I.P., Singh S., Dhawan V. (2020). Effect of alkali treatment on mechanical properties of jute fiber-reinforced partially biodegradable green composites using epoxy resin matrix. Polym. Polym. Compos..

